# Dissociable neural mechanisms underlie currently-relevant, future-relevant, and discarded working memory representations

**DOI:** 10.1038/s41598-020-67634-x

**Published:** 2020-07-08

**Authors:** Elizabeth S. Lorenc, Annelinde R. E. Vandenbroucke, Derek E. Nee, Floris P. de Lange, Mark D’Esposito

**Affiliations:** 1 University of California, Berkeley, CA USA; 2 University of Texas at Austin, Austin, TX USA; 3 Donders Institute for Brain, Cognition and Behavior, Radboud University, Nijmegen, The Netherlands; 4 Florida State University, Tallahassee, FL USA

**Keywords:** Attention, Perception

## Abstract

In daily life, we use visual working memory (WM) to guide our actions. While attending to currently-relevant information, we must simultaneously maintain future-relevant information, and discard information that is no longer relevant. However, the neural mechanisms by which unattended, but future-relevant, information is maintained in working memory, and future-irrelevant information is discarded, are not well understood. Here, we investigated representations of these different information types, using functional magnetic resonance imaging in combination with multivoxel pattern analysis and computational modeling based on inverted encoding model simulations. We found that currently-relevant WM information in the focus of attention was maintained through representations in visual, parietal and posterior frontal brain regions, whereas deliberate forgetting led to *suppression* of the discarded representations in early visual cortex. In contrast, future-relevant information was neither inhibited nor actively maintained in these areas. These findings suggest that different neural mechanisms underlie the WM representation of currently- and future-relevant information, as compared to information that is discarded from WM.

## Introduction

Working memory (WM) is a cognitive ability that allows one to hold information in mind while processing other information relevant to the task at hand. Most WM research has focused on mechanisms supporting the maintenance of information that is within the focus of attention^1–4^. These studies have lent support to the ‘sensory recruitment’ hypothesis^1,5,6^, which proposes that the same brain regions that process information during perception are involved in the maintenance of task-relevant mnemonic representations. Consistent with this hypothesis, stimulus-specific activity has been observed in early visual cortex during the delay period in visual WM tasks^1–4^. However, in daily life, WM does not exclusively consist of information in the focus of attention. Rather, information that is not currently in use, but may be required later, must also be concurrently maintained, and information that is no longer relevant must be discarded. An outstanding question is whether similar ‘sensory recruitment’ mechanisms support the maintenance of unattended (but future-relevant) information, and the removal of unattended, future-*ir*relevant information.

Recent work seeking to characterize attended and unattended WM representations has typically found that perceptual regions contain active representations of attended, but not unattended, mnemonic information^7–10^. It has therefore been suggested that un-cued, future-relevant WM items might be maintained in an activity-silent format, perhaps through a change in the strength of synaptic connections^11–13^. Indeed, when the system is activated by a non-specific visual stimulus or a single pulse of transcranial magnetic stimulation (TMS) during a memory delay, category information about an un-cued WM item can be transiently decoded with electroencephalography (EEG)^14–16^. This implies that this information may be represented in perceptual regions in a silent code that can only be decoded when it is activated, either through a shift in internal attention or an external perturbation like TMS. Further, other recent work has found representations of both cued and un-cued information in regions of frontal and parietal cortex, suggesting that these regions may play a complementary role in the (perhaps lower-resolution) coding of unattended future-relevant information^10^.

Behavioral evidence shows that un-cued future-relevant information does not influence memory retrieval times^17,18^, indicating that such items do not compete with attended items during retrieval. Nevertheless, un-cued future-relevant items can be recalled just as accurately as attended information^18^. These data indicate that the neural coding scheme for un-cued future-relevant information must strike a balance between accessibility and non-competition. Given that active neural firing could lead to competition between cued and un-cued representations^19–21^, synaptic changes may allow future-relevant information to be retained without interfering with currently-relevant representations. In addition, future-*ir*relevant information (which is discarded from WM) can still cause proactive interference^22–25^, indicating that it must also persist in some way. It is plausible that such discarded WM representations may also be maintained by synaptic connections. However, non-specific visual stimulus- or TMS-evoked reactivation has been found only for un-cued, future-*relevant* WM items, and not for discarded items^14–16^. In contrast, a recent experiment by van Loon et al.^26^ found graded, but above-chance, evidence for VWM delay-period representations for currently-relevant, future-relevant, and future-irrelevant targets of an upcoming visual search task^26^. Interestingly, van Loon et al. observed a transformation of future-relevant target representations *during* the search task, such that they became anti-correlated with currently-relevant representations. However, such a transformation was not observed for future-irrelevant (discarded) information.

In the current study, we sought to investigate the neural representations underlying each of these three mnemonic states: cued items that are in the focus of attention, un-cued items that are relevant for future use, and un-cued items that are irrelevant for future use. Participants were instructed to remember the precise orientations of sinusoidal gratings; low-level stimuli known to be processed (and maintained) in early visual cortex^27,28^. Using these stimuli, we could investigate whether sensory recruitment can account for the three WM states. We measured fMRI blood-oxygen-level-dependent (BOLD) responses and employed classical multivoxel pattern analysis (MVPA) orientation classification techniques. In addition, we used an inverted encoding model (IEM) approach to link a specific model of the presumed underlying population-level neural representations to the observed BOLD data^29,30^. This also allowed us to test different plausible neural mechanisms underlying the different WM states using computational modeling of simulated data based on our empirical findings.

## Results

**Figure 1:**
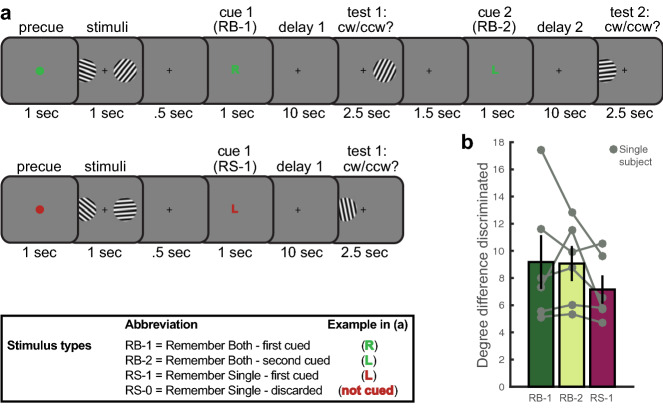
(**a**) Experimental design. Upper panel depicts a trial in which participants remembered both orientations, and were sequentially tested on the two orientations. Lower panel depicts a trial in which participants initially remembered both orientations, but upon cueing maintained one item and discarded the other. Abbreviations are used here and in further figures. Note that the order of cueing the left or right stimulus was counterbalanced within RB trials as well as between all trials. (**b**) Behavioral performance was measured by the difference in degrees between the memory and test orientation that participants discriminated at a 75%-correct threshold. Lower difference discrimination represents better memory.

We collected BOLD fMRI data while six participants performed a task in which they had to remember the orientations of two lateralized gratings (Fig. 1a). There were two trial types: Remember Both (RB) and Remember Single (RS), indicated by the color of the pre-cue. In the RB condition, a green letter was presented after offset of the memory items indicating which orientation should be recalled first (L for Left, R for Right; “RB-1” items). During the first delay period, the second orientation had to be maintained as well, since it would be probed for recall later in the trial (“RB-2” items). After the first delay period, participants indicated whether a test orientation presented at the first cued location was tilted clockwise or counter clockwise compared to the RB-1 memory orientation. The RB-2 item was then cued and tested after a second delay period (thereby becoming attended to during the second delay period). On RS trials, a red letter indicated which orientation was relevant (“RS-1”), and this simultaneously implied that the other orientation could be discarded (“RS-0”).

### Behavior

Across all runs, participants could discriminate orientation differences equally well between first- and second-cued items on Remember Both trials (RB-1 vs. RB-2, Wilcoxon signed rank *p* = 1.0; Bonferroni-corrected *α* = 0.017; Fig. 1b). This suggests that participants successfully maintained RB-2 items in WM for later use. Discrimination performance was marginally better for the cued item on Remember Single trials than the first-cued item on Remember Both trials (RB-1 vs. RS-1, Wilcoxon signed rank *p* = 0.06), but did not differ between RB-2 and RS-1 items (Wilcoxon signed rank *p* = 0.16).

To further examine behavior once staircasing and learning effects had stabilized, we repeated the behavioral analyses on the data from the final MRI session alone, and found that orientation discrimination was numerically most precise for RS-1 items, followed by RB-1 and then RB-2 items, although no comparisons passed the Bonferroni-corrected α = 0.017 (Wilcoxon signed rank tests, RS-1 vs. RB-1 *p* = 0.03, RB-1 vs. RB-2 *p* = 0.03, RB-2 vs. RS-1 *p* = 0.03; Supplementary Fig. S1).

### Univariate whole brain analysis

**Figure 2:**
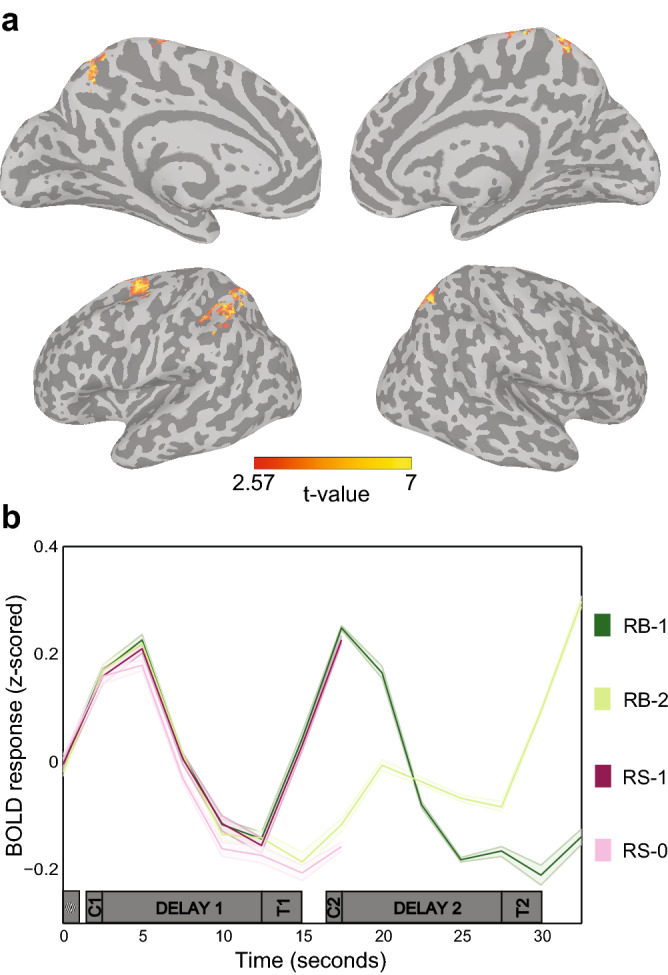
(**a**) Univariate whole brain analysis of mean activity during the first delay period, voxel-wise corrected at *p* < 0.05 with a cluster correction of *p* < 0.001. (**b**) Mean BOLD signal (z-scored) in early visual cortex (averaged over hemispheres) for the four stimulus types. Gray bars depict trial timings. Shaded areas represent between-subject standard error. See Fig. 1 for abbreviations.

Univariate whole brain analyses showed that parietal and superior frontal areas exhibited elevated activity during the first delay period (Fig. 2a), consistent with previous WM studies^4,31,32^. No significantly elevated delay activity in early visual cortex was found (Fig. 2b)^1–3,32^, even at lower statistical thresholds (Supplementary Fig. S2).

### Classification and reconstruction analyses in early visual cortex

To investigate how currently-relevant and future-relevant or discarded memory representations were processed, we used both a logistic regression multivoxel pattern analysis^7,8^ (classification) and an inverted encoding model approach^29,30^ (reconstruction). The IEM allowed us to conduct simulations of potential neural mechanisms underlying the reconstructed memory representations. Based on previous work demonstrating that mnemonic representations for orientation are maintained through stimulus-specific delay-activity in visual cortex^1–4^, we first analyzed the BOLD signal for the four stimulus types (RB-1 and RB-2, RS-1 and RS-0) in a predefined visual ROI (combined V1–V3). Stimuli were classified and reconstructed during five task periods: stimulus presentation, first delay, first test for both RB and RS trials, and second delay and second test for the RB trials only (see Fig. 2b and “Methods”).

Previous studies have used BOLD signal from either the stimulus presentation period or the delay period as basis for training classifiers to test for stimulus-specific delay activity^3,4,7^. If the same population of neurons both processes and maintains a stimulus orientation, one would expect to find accurate decoding and reconstruction when training on stimulus presentation and testing delay period representations^2^. However, if one expects that visual cortex remains involved in the maintenance of stimulus orientation, but that these representations change over time^12^, training models on the stimulus presentation period would result in inaccurate delay period decoding/reconstruction performance. To maximize our ability to find evidence for the unattended WM representations, we therefore trained the classifier and IEM on both the stimulus presentation period and the first delay period of the task in separate analyses. For both methods, training was performed on all trials together whereas testing was performed on each stimulus type separately. Both training and testing were performed for each hemisphere separately, and test results were then averaged according to stimulus type.

### Orientation classification

For the classification using logistic regression, orientations were binned into three ‘base’ orientations (see “Methods”). We assessed classifier performance by subtracting the classifier evidence for the orientation that was not presented during that trial from the evidence for the tested orientation. Thus, positive values represent evidence in favor of the tested orientation, while negative values represent suppression of the tested orientation (i.e. evidence in favor of the not-presented orientation).

**Figure 3:**
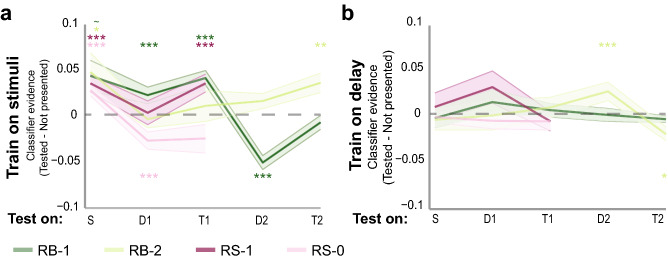
Early visual cortex decoding analyses. Classification after training on the stimulus presentation period (**a**) and the first delay period (**b**). Classifier performance is measured by subtracting the evidence for the not-presented category from the tested category on each separate trial (see Fig. 1 for abbreviations). Shaded areas represent between-subject standard error. *p < 0.05, **p < 0.01, ***p < 0.001. For an overview of classification per time point, see Supplementary Fig. S4; for classification performance per participant, see Supplementary Fig. S5. For raw classifier evidence values, in which the not-presented category has not been subtracted, see Supplementary Fig. S3.

Using a classifier trained on the stimulus presentation period (“stimulus-period classifier”) (Fig. 3a), we found significant positive evidence for the RB-1 item (*p* < 0.001) during the first delay period [but not RS-1 (*p* = 0.886)]. Using a classifier trained on the first delay period (“delay-period classifier”) (Fig. 3b), both the RB-1 (*p* = 0.218) and RS-1 (*p* = 0.312) item showed numerically positive evidence, although this was not statistically reliable across participants. While the RB-2 item could not be decoded during the first delay period using stimulus-period (*p* = 0.530) or delay-period (*p* = 0.496) classifiers, this item could be decoded with a delay-period classifier when it became currently-relevant in the second delay period (*p* < 0.001). These results are consistent with previous research demonstrating that only currently-relevant, and not un-cued, future-relevant information can be decoded from WM^1–4,7,8^ within sensory cortices.

From these analyses, it is unclear what distinguishes the representation of information that will become relevant in the future from information that will never be relevant, and can thus be discarded. To examine this in more detail, we examined classifier evidence for the RS-0 item, which was never probed. In contrast to the RS-1 and RB-1 items, there was significantly *negative* relative evidence for the orientation of the RS-0 item using a stimulus-period classifier (*p* < 0.001). Because the classifier was trained and tested on separate trial epochs, such negative evidence is plausible (and meaningful) in this analysis (note that negative evidence would be unlikely and harder to interpret for a within-epoch classifier, because the same data alternately serves as training and testing examples across cross-validation iterations). Therefore, the negative evidence found here shows that the activity pattern evoked in the contralateral hemisphere when a stimulus is no longer relevant is opposite from the pattern evoked for that stimulus during initial processing.

However, one possibility for the negative evidence observed for the RS-0 item may be incomplete encoding, since the item cue closely followed the encoding period. To examine this possibility, we examined evidence for the RB-1 item in the *second* delay, when the item is no longer relevant and will not be relevant again. If negative evidence for the RS-0 item were due to incomplete encoding, then the RB-1 item should have no evidence during the second delay, rather than negative evidence. However, if negative evidence reflects once-relevant memory information being no longer relevant, we would expect that there would be negative evidence for the RS-1 item during the second delay, as we found for the RS-0 item during the first delay. Consistent with the latter possibility, this analysis revealed significant negative evidence for the RB-1 item during the second delay period (*p* < 0.001) when training on the stimulus presentation. This suggests that the representation underlying never-relevant information is different from the representation underlying information that is currently un-cued, but will be relevant in the future.

**Figure 4:**
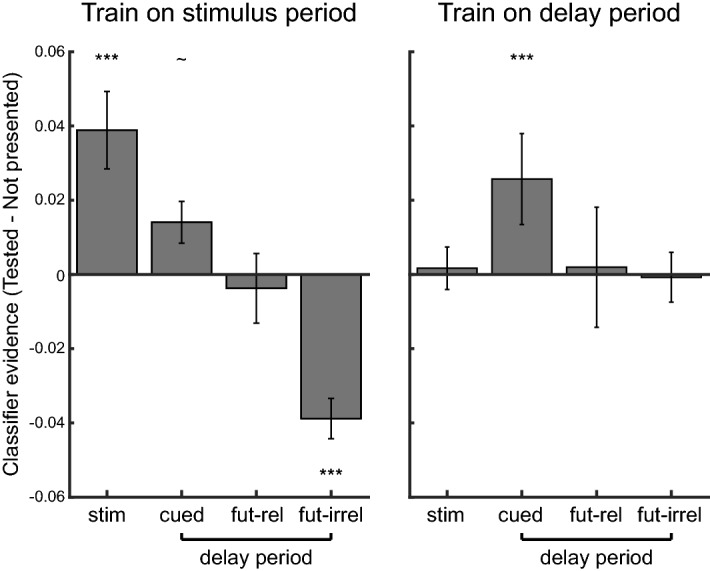
Summary of the ROI analyses when training the classifier on the stimulus period (**a**) and the first delay period (**b**). Evidence is summarized over all stimulus presentations (stim), all cued items (cued), all un-cued future relevant items (fut-rel) and un-cued, future irrelevant items (fut-irr). Error bars denote between-subject standard errors.

To summarize the findings from the ROI analyses, we averaged the different stimulus types into the four tested phenomenological states: the presentation of the stimuli (“stim”), the cued items (“cued”: RB-1 and RS-1 during delay 1; RB-2 during delay 2), the un-cued future-relevant item (“fut-rel”: RB-2 during delay 1) and the un-cued future-irrelevant items (“fut-irrel”: RS-0 during delay 1 and RB-1 during delay 2). As can be seen in Fig. 4 (left plot), the stimulus-period classifier evidence values differed across the three different types of delay-period representations (one-way non-parametric repeated measures ANOVA (see “Methods”): *F*(2,10) = 11.249, *p* = 0.001), with positive evidence for cued items (*p* = 0.080), no evidence for un-cued future-relevant items (*p* = 0.530), and significantly negative evidence for un-cued future-irrelevant items (*p* < 0.001). Follow-up paired comparisons indicated that the most reliable difference was between the cued and un-cued future-irrelevant items (*p* < 0.001) (cued vs. un-cued future-relevant *p* = 0.260; un-cued future-relevant vs. un-cued future-irrelevant *p* = 0.076). In addition, when training on the first delay period (Fig. 4, right plot), we found reliable positive evidence for cued items (*p* < 0.001), but not perceived stimuli (*p* = 0.572), un-cued future-relevant items (*p* = 0.496), or un-cued future-irrelevant items (*p* = 0.902), although the difference across WM states was less pronounced (one-way non-parametric repeated measures ANOVA: *F*(2,10) = 2.810, *p* = 0.105). However, as discussed above, this delay-period analysis had less power to reveal negative evidence for discarded representations, because the training and testing occurred within a single trial epoch. Thus, in this analysis, the training examples included a mix of cued, un-cued, and discarded items, making it unlikely that the discarded item representations in the test set would show reliably opposite patterns from the training examples. Indeed, when we trained instead on only the cued items during delay 1, we found a hint of negative evidence for the discarded items, albeit less so than when we trained on the stimulus representations (see Supplementary Fig. S6).

Finally, the pattern of results when training on the stimulus period looked quite different compared to the results when training on the first delay period (Fig. 4); classification was most successful *within* task epoch, compared to when the classifier was trained and tested *between* stimulus presentation and memory delay periods. A non-parametric 2 (classifier training epoch: stimulus-period vs. delay-period) × 2 (state: stimulus vs. cued delay) repeated measures ANOVA confirmed an interaction between classifier training and testing epoch (*F*(1,5) = 11.014, *p* = 0.017). Training on the stimulus period resulted in better decoding when a stimulus was present, while training on the delay period resulted in better decoding of cued items during the delay periods. This is in line with previous findings that, while there is considerable generalizability between stimulus-evoked and attended WM representations, these representations are not identical^2^.

**Figure 5:**
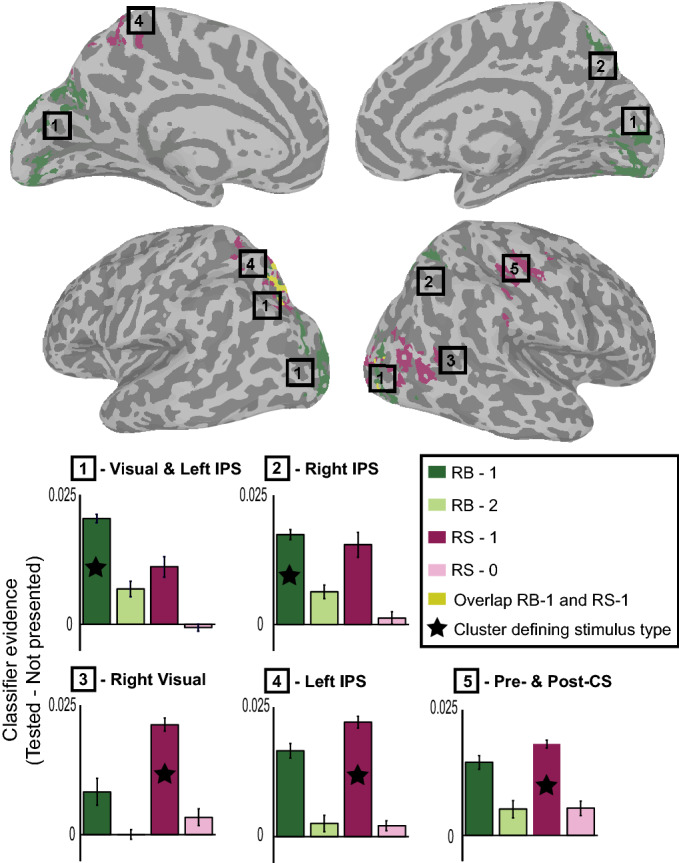
Searchlight analysis for the first delay period. Voxel-wise correction of *p* < 0.05 with a cluster correction of *p* < 0.001. Clusters were determined per stimulus type (see Fig. 1 for abbreviations), and graphs depict the evidence values for each stimulus type in each cluster. The star indicates the condition within which the cluster was identified, and is therefore only presented for reference. Error bars represent between subject error. *IPS *intraparietal sulcus, *CS *central sulcus.

We were unable to decode un-cued, future-relevant items from the early visual cortex ROI. To explore whether other brain regions were involved in the maintenance of un-cued, future-relevant items, we conducted a searchlight analysis^33^. This analysis systematically tests spherical spatial patterns throughout the whole brain. Given that the classification analysis using the delay period as the training period produced the most robust decoding of WM representations in early visual cortex, we used this task epoch for classifier training in the searchlight analysis as well. In addition, we did not assume that WM representations were confined to the retinotopic locations of the presented stimuli; the classifier was trained on the cued orientation, regardless of the visual hemifield in which it was presented. For each trial in the test set, we subtracted the evidence for the not-presented orientation from the evidence for the cued and the un-cued orientations. Thus, positive values represent evidence in favor of the tested orientation, while negative values represent evidence in favor of the not-presented orientation (i.e. suppression of the tested orientation category). For both RS-1 and RB-1 items, positive evidence for a mnemonic representation was found in visual cortex and intraparietal sulcus (IPS; Fig. 5). In addition, the pre- and post-central sulci showed positive evidence mainly for the RS-1 item. For visualization, we depict the evidence for each memory type separately in each cluster, illustrating that currently-relevant items for both the RS and RB trials are represented in pre- and post-central sulci, IPS and visual cortex bilaterally. No significant clusters were found for RB-2 items during the first delay period. Evidence for the representation of the RS-0 item was also investigated using the whole brain searchlight analysis described above. No additional brain regions showed positive or negative evidence for the RS-0 item when the classifier was trained on RB-1 and RS-1 items.

### Inverted encoding model analysis and computational modeling

The negative classifier evidence found for the un-cued, future-irrelevant items suggests that this representation might be suppressed when it is discarded from WM. To investigate the potential mechanisms underlying suppression of these items, we used an IEM orientation reconstruction approach. This allowed us to estimate population level feature-selective tuning functions^34^. The IEM we used was based on the assumption that a visual cortex neuron orientation response follows a tuning curve with peak firing at a preferred orientation, which across the entire population can be observed at the level of fMRI voxels^29,35^. We utilized this characteristic to reconstruct the orientation response curve for the population of voxels in visual cortex, giving us information about the most likely orientation being seen/remembered by the participant. These results were used to inform simulations regarding the potential mechanisms supporting WM states.

To reconstruct orientation response curves, we first created a basis set of hypothetical orientation channels that reflected the expected orientation response profiles of visual cortex^29,35^. We then trained on a subset of the data to calculate, for each voxel, the weights on these orientation channels that best described its BOLD response to each presented orientation (see “Methods”). Next, using these weights and the measured BOLD responses in all left-out trials, we calculated the responses of each of the hypothetical orientation channels. We then created trial-wise reconstruction functions by calculating a weighted sum of the hypothetical orientation channels, weighted by the calculated channel response magnitudes. The resulting orientation response curves from each trial were re-centered so that 0 corresponded to the actually presented/remembered orientation, and then averaged according to stimulus type as in the classification analysis.

**Figure 6:**
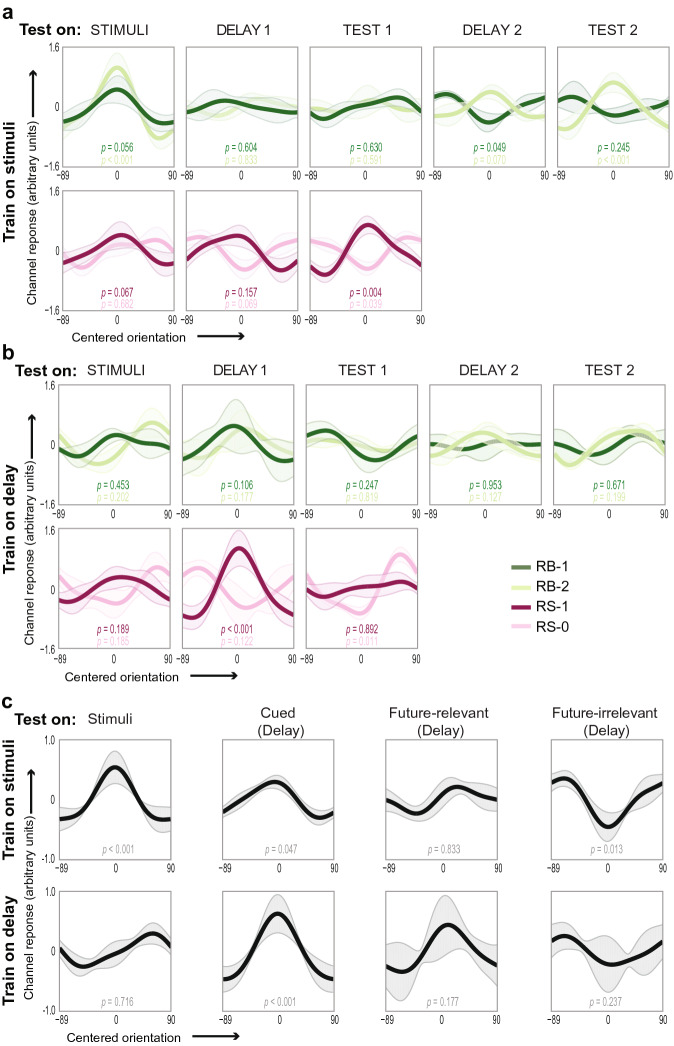
Early visual cortex IEM reconstruction analyses. These results show a similar pattern as the classification (Fig. 3) both when trained on the stimulus period (**a**) and the delay period (**b**; see Fig. 1 for abbreviations). **c** Evidence is summarized over all stimulus presentations (stim), and delay representations for all cued items (cued), un-cued future relevant items, and un-cued, future irrelevant items. Shaded areas represent between-subject standard error. *p < 0.05, **p < 0.01, ***p < 0.001. For reconstructions on each individual time-point, see Supplementary Fig. S6; for IEM reconstructions per participant, see Supplementary Fig. S7.

The results from the IEM closely matched the classification results (Fig. 6), both with a model trained on the stimulus presentation period (Fig. 6a) and with a model trained on the first memory delay (Fig. 6b). Perhaps most informative, though, are the summary reconstructions (Fig. 6c), collapsed according to item status as in Fig. 4. An IEM trained on the stimulus perception period allowed successful reconstruction of perceived stimuli (*p* < 0.001) and cued (*p* = 0.047) but not un-cued future-relevant (*p* = 0.833) stimuli during the WM delay. In contrast, reconstructions for un-cued future-irrelevant items were inverted (*p* = 0.013), corresponding to the negative classifier evidence observed in the classification analysis. As in the classification analysis, meaningfully negative evidence can occur in this analysis, because model training and testing was performed in separate trial epochs. An IEM trained on the first delay period allowed successful reconstructions of cued stimuli during the delay (*p* < 0.001), but not perceived stimuli (*p* = 0.716), or un-cued items during the memory delay, regardless of whether they were future-relevant (*p* = 0.177) or future-irrelevant (*p* = 0.237). This close correspondence between the IEM reconstructions and the classification results indicated that an explicit IEM that linked the presumed underlying neural representations to the BOLD data could be used to explore the neural mechanisms underlying the maintenance and removal of orientation information in WM.

Attention^36^ and retaining information in WM can alter the receptive field properties of neurons^37^. Such changes in receptive field properties can give rise to negative readouts of previously attended information^38^, which result in biases in subsequent perception and/or memory^38,39^. To examine whether receptive field property changes could lead to the observed representational suppression, we created a computational model wherein the gain, receptive field width and or/receptive field center of neurons could change post-encoding. We created simulated data using the basis set of channels as used in the IEM reconstructions (see “Methods”). The stimulus phase of the simulated data was used to train an IEM. Next, a simulated test data set was created wherein parameters controlling the gain (γ), receptive field width (μ), and receptive field centers (δ), along with memory strength (φ) were varied. These parameters were fit to the experimental data averaged over stimulus types (averaged as in Fig. 4). Separate fits were performed for data tested during the stimulus phase, and during the delay phase for each of the cued, un-cued future-relevant, and un-cued future-irrelevant representations. Since there was no evidence of reliable ipsilateral stimulus/memory representations in the experimental data (Supplementary Fig. S9), only contralateral stimulus representations were simulated.

**Figure 7:**
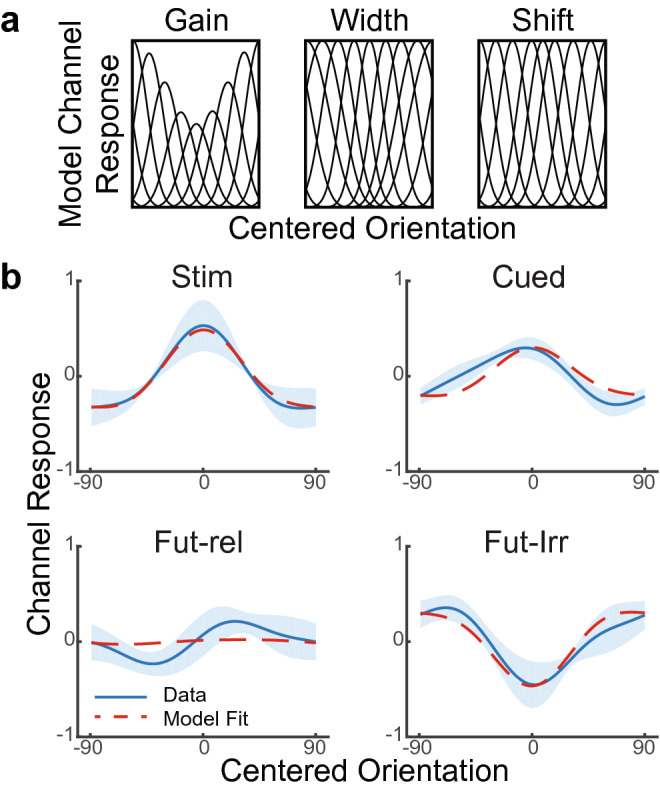
(**a**) Different modulatory effects were modeled to simulate retention and suppression. Gain modulations (γ) reduced the amplitude associated with channels near the represented orientation. Width (μ) modulations increased the width of channels far from the represented orientation. Shift (δ) modulations shifted channels towards the represented orientation. (**b**) Simulations of retention and suppression. Blue lines depict averaged data. Evidence is summarized over all stimulus presentations (stim), all cued items (cued), all un-cued future relevant items (fut-rel) and un-cued, future irrelevant items (fut-irr). Shaded area denotes between-subject standard error. The red dotted line shows the modeled data using the modeling parameters that best fit the data (Stim: φ_test_ = 0.98, γ, μ, δ = 0; Cued: φ_test_ = 0.61, γ, μ, δ = 0; Fut-Rel: φ_test_ = 0.03, γ, μ, δ = 0; Fut-Irr: φ_test_ = 0.49, γ = 0.95; μ, δ = 0).

First, we tested the ability of the model to reproduce the stimulus phase data. Here, the training data should have the same properties of the test data (i.e. no modulations, φ = 1). Indeed, this was observed (φ_test_ = 0.98, γ, μ, δ fixed to 0; R^2^ = 0.99; Fig. 7a). Model comparison using a nested model approach revealed that freeing parameters did not improve the fit (all *p* > 0.15). Similarly, the cued representation could be fit without modulations, but with a reduction in memory strength (φ_test_ = 0.61, γ, μ, δ fixed to 0; R^2^ = 0.72; Fig. 7b) and freeing parameters did not improve the fit (all *p* > 0.2). In both of these cases, the simulated data tended to be within a single standard error of the actual data (average s.e. for stimulus = 0.21, average s.e. for cued = 0.94). The un-cued future-relevant representation could also be fit within one standard error without modulations (average s.e. = 0.76). In this case, the fit parameter for memory strength was essentially 0 (φ_test_ = 0.03, γ, μ, δ fixed to 0; R^2^ = 0.71), resulting in a flat reconstruction. Freeing parameters did not improve the fits (model comparisons: all *p* = 1).

Finally, the un-cued future-irrelevant representation could not be satisfactorily modeled by varying memory strength alone (average s.e. = 1.59; φ_test_ = 0.00, γ, μ, δ fixed to 0; R^2^ = 0.13) with the reconstruction produced by the simulation negatively correlated with the data (r = − 0.35). The un-cued future-irrelevant representation was better fit by freeing either gain (*F*(1,16) = 55.91, *p* < 10^–6^) or width modulation parameters (*F*(1,16) = 41.07, *p* < 10^–5^), but not the shift modulation parameter (F(1,16) = 0.23, *p* > 0.6). We next explored whether modulations alone could fit the data by fixing memory strength to 0. Although gain and memory strength trade-off to some degree (i.e. the gain modulations act inversely to memory strength), the data were better fit by freeing both parameters compared to a model that fixed memory strength to 0 (F(1,16) = 8.04, *p* < 0.05; φ_test_ = 0.49, γ = 0.95; μ, δ fixed to 0; R^2^ = 0.90). On the other hand, the memory strength parameter was unnecessary for a model including width modulations (nested model comparison *p* = 1), and the same width modulation parameter (μ = 1.00) was observed whether memory strength was free to vary or fixed to 0 (φ_test_, γ, δ fixed to 0; R^2^ = 0.88).

Given that the negative reconstruction of un-cued future-irrelevant representations could be fit assuming width modulations and no memory strength, this opens the possibility that the negative reconstruction is an after effect of width modulations rather than an active mechanism. That is, if one assumes that width modulations occur as a consequence of perception^39^, then what appears to be a suppression of an irrelevant representation could be a consequence of altered tuning coupled with ceasing active maintenance of the irrelevant representation. In this case, one would expect similar width modulations to be observed in other memory states. Although adding width modulations did not improve model fits for the stimulus phase, cued item, and un-cued future-relevant item, we nevertheless examined width modulation parameters for these items to see if consistent width modulations might be present in all states. However, letting the width modulation parameter, μ, freely vary led to inconsistent estimates across states (stimulus: μ = − 0.18; cued: μ = − 1; un-cued future-relevant: μ = 0.45) none of which resembled the fitted parameter for un-cued future-irrelevant items (μ = 1). Furthermore, the fitted parameter for un-cued future-irrelevant items was at the extreme of our range, and width modulations of such a magnitude may not be biophysically plausible.

Given that the memory strength + gain modulation model and width modulation model are not nested, a nested model comparison could not be performed. However, the memory strength + gain modulation model produced a slightly better adjusted R^2^ than the width modulation model (0.89 vs. 0.87) and had a lower average s.e. (0.52 vs. 0.69). Hence, the best fit to the data was explained by a model that surprisingly included some memory activity related to the un-cued future-irrelevant representation coupled with gain suppression.

## Discussion

The results of the current study are congruent with previous findings demonstrating that early visual cortex maintains a representation of cued WM items^1–4^. In addition, both bilateral IPS and the right pre- and post-central sulci exhibited above-chance decoding of orientations maintained in WM. These findings add to the growing body of evidence that visual WM representations are maintained in a distributed set of brain regions not confined to visual cortex^4,10,40–43^.

However, WM is not only required for the maintenance of currently-relevant, cued information. Rather, it is also critical that WM simultaneously maintains information that is not currently relevant but will be needed in the future, and discards no-longer-needed representations. In this study, we investigated how unattended future-relevant and unattended future-irrelevant WM items are represented in visual cortex. We manipulated attention by cueing one out of two stimuli during an initial delay period. The second stimulus was un-cued, but either relevant for use after the first delay period, or never relevant after the first delay period. Therefore, un-cued future-relevant items needed to be maintained in WM, but not in the focus of attention, while un-cued items that would never be relevant did not have to be maintained in WM. We found that an un-cued, future-relevant item could not be decoded from activity patterns in visual cortex, as has been found previously^7–10^. There is, however, an interesting recent exception to this finding: an analog of object-based attention seems to hold in WM, in that un-cued future-relevant information remains measurable if other features of the same object are currently-relevant^44,45^.

In contrast, we found that an item that was discarded from WM—either immediately after encoding or after being used in the current trial—elicited a negative IEM reconstruction and decoding evidence that was less than that of a stimulus that was not presented. The negative decoding evidence for discarded WM items suggests that representations in visual cortex associated with future-irrelevant features are suppressed. It is important to note that the decoding evidence was negative compared to a stimulus that was not presented in the trial, the negative evidence only occurred in specific trial periods, and it was distinct from the observed lack of evidence (either positive or negative) for the un-cued, future-relevant item. This finding adds to an emerging body of evidence for suppressive effects in visual WM and in other related cognitive domains. First, Ester et al.^4^ observed inverted IEM reconstructions for un-cued (future-irrelevant) orientations in VWM in some visual, parietal, and frontal regions, although this cannot unequivocally be interpreted as evidence for suppression because model training and testing were both performed within a single task epoch (see “Methods”). Second, Sahan et al.^45^ recently found some evidence for inverted IEM reconstructions for the motion direction of colored moving-dot stimuli in WM when this feature was un-cued and future-irrelevant. Perhaps meaningfully, such an inversion was most often found in the presence of an active representation for cued, currently-relevant information. When a direction of motion was cued to be tested a second time, an active direction representation was no longer observed for the cued information, nor was an inverted representation seen for the un-cued future-irrelevant direction (see Sahan et al.^45^, Fig. 6). The authors postulated that these observed inverted reconstructions could be due to active suppression or recoding of irrelevant WM representations, potentially in order to reduce interference with currently-relevant information. Finally, van Loon et al.^26^ decoded the category of object targets held in VWM in preparation for a visual search task. In contrast to the current results, during the unfilled delay period prior to the visual search (most similar to our blank delays), decoding was above chance, regardless of whether an item was currently- or future-relevant, or could be discarded. Instead, a suppression-like effect was found during the visual search task, in that the representational patterns for un-cued future-*relevant* items became anti-correlated with those for currently-relevant items^25^. Interestingly, again in contrast to our results, future-*irrelevant* information did not show such an inversion. While it is difficult to directly compare the current findings to these previous results, it is possible that the potential for interference among WM representations was lower in the previous work (a cow, dresser, or skate vs. flower) compared to our stimuli that varied along a single visual feature (orientation). This may have necessitated an active suppression of irrelevant information during the WM delay of our task, while similar mechanisms may only have been required during active visual search in the previous study.

Also in line with the current findings, previous studies on attention show that decoding accuracy for items that are selectively ignored is lower compared to neutral items that are neither attended nor ignored^46^, and lower compared to stimuli that were not present during the trial^47^. In addition, the suppression for future-irrelevant WM items that was observed in this study may be linked to competition in long-term memory. For example, when attention is switched among items during WM maintenance, larger competition between items leads to worse long-term memory for these items^48^. Also, if one of two competing memory items is retrieved after a delay period, this leads to subsequent forgetting of the other memory item that was not retrieved^49,50^.

Our computational modeling of simulated data suggests that the negative reconstruction for the discarded WM representation in visual cortex can result from a change in population tuning curve properties. When the amplitude or width of the channel response decreased as a function of distance to the discarded orientation, the simulations produced suppression of the discarded WM item. Behaviorally, it has been shown that such mechanisms produce biases in the perception of subsequent stimuli^39,51^. It is possible that suppression of discarded WM representations could also be a neural mechanism to help mitigate proactive interference effects^23,45,52,53^. By contrast, our simulations could not reproduce suppression of no longer relevant WM representations by shifting the preferred orientation for each channel response towards the discarded orientation. Neurons in the visual system have been demonstrated to display a shift of their receptive fields towards relevant information^36^, and such shifts persist even after information is no longer relevant, producing biases in neural readouts and behavior^38^. However, in our simulations we found that such shifts were insufficient to produce suppression under the model assumptions.

Based on our BOLD data and computational modeling of simulated data, we infer that a mechanism of orientation suppression operates on the neural population level when an orientation needs to be dropped from WM. This inference is in line with orientation suppression effects after visual adaptation^54^, in which neuronal orientation tuning is altered due to mutual inhibition^55^. Additionally, monkey physiology data shows that suppression might serve as a mechanism during similar WM tasks. For example, when monkeys need to saccade to a memorized location, inhibited delay period activity has been observed for the orthogonal location^56^. Inhibition in these cases is thought to serve as a mechanism through which a saccade to a certain location is suppressed^57,58^. Such active suppression is in contrast to another proposed mechanism, in which information could instead be dropped from WM by severing the binding between an item and its spatiotemporal context within the trial^59^.

Alternatively, rather than indicating active suppression of a no-longer-needed representation, it could be that the suppression of the un-cued, future-irrelevant item was caused by an after-effect of perceiving an orientation. Our computational modeling showed that the reconstruction of the un-cued, future-irrelevant item could be fit by broadening the tuning of channels away from the un-cued, future-irrelevant item coupled with the absence of memory activity. If one assumes that such tuning occurs as a consequence of perception^39^, then a suppressed representation may be read out from the altered tuning coupled with no representation-related activity. However, one would expect similar tuning modulations to be observed in all conditions following perception, which we did not find in simulations. Active suppression via gain modulations provided a better explanation of the data.

Orientation decoding and reconstruction success differed when the classifier and IEM were trained on perception versus memory maintenance. When the models were trained on initial stimulus perception, decoding and reconstruction were better for trial periods in which stimuli were present on the screen (Stimulus, Test 1 and Test 2) than they were during the memory delay periods (Delay 1 and Delay 2). Conversely, models trained on the first memory delay were more successful at decoding and reconstructing during the first and second memory delay periods than any of the stimulus presentation periods. This suggests that the representation of a stimulus partly changes from perception to memory; although visual cortex is still involved, the pattern of activity that codes for the representation is different^15,60,61^. Therefore, WM might not be characterized by persistent activity that exactly mimics stimulus processing, but rather as a more dynamic process that changes throughout the maintenance period^12^.

In this study, we did not find positive evidence for the un-cued, but future-relevant WM item in a pre-defined visual cortex ROI, or outside of visual cortex using a searchlight procedure. Given that we found positive evidence for cued items, and negative evidence for discarded items, in visual cortex, this suggests that un-cued, future relevant items are represented in a manner distinct from both. That is, they are not maintained in activity similar to cued items, but neither are they suppressed. In addition, when previously un-cued items were subsequently cued and attended to, they could be decoded from visual cortex, suggesting that these future-relevant representations had been maintained in visual cortex. Alternatively, it is possible that un-cued, future-relevant WM information is maintained in a different, perhaps more abstract^10,62^, form in another brain region, and is only reinstated in visual cortex when cued. This could be beneficial for protecting the visual memory trace from subsequent visual input^40^ (but see^63^). However, if this were the case, we would expect a qualitative difference between cued items continuously maintained in visual cortex and un-cued items subsequently reinstated in visual cortex. Since we found that the second cued representation was comparable to the representation during the first delay period, our data does not support this alternative. Whether the un-cued, future-relevant item is maintained by active firing in visual cortex that is sparse enough to be difficult to detect using fMRI, or whether it is maintained by an inactive mechanism, such as a change in synaptic weights^11,12,64,65^ remains to be elucidated. Regardless, our data show clear evidence that such information is retained in a qualitatively distinct manner from no-longer-relevant information.

## Methods

### fMRI acquisition

Six volunteers (2 male, age 19–36) participated in this experiment for monetary compensation. All subjects had normal or corrected-to-normal vision and were screened for possible risk factors precluding participation in MRI experiments. All procedures were approved by the UC Berkeley Committee for the Protection of Human Subjects and performed in accordance with UC Berkeley MRI participant guidelines. Volunteers gave written informed consent for participation.

Each participant completed four 2-h MRI sessions. Scanning was performed at the UC Berkeley Henry H. Wheeler, Jr. Brain Imaging Center with a Siemens TIM/Trio 3 T MRI scanner with a 32-channel head coil. A high-resolution T_1_-weighted anatomical image (TR: 2,300 ms, TE: 2.98, FOV: 80 × 80 × 160, flip angle: 60°) was recorded during each participant’s first scan session. Functional MRI data were obtained using a T_2_-weighted echoplanar imaging (EPI) sequence (TR: 2,500 ms, TE: 29, voxel size: 2.5 × 2.5 × 2.5 mm, interslice gap: 0.5 mm, 37 slices descending acquisition, FOV: 80 × 80 × 200). Functional data for retinotopy and localization purposes were collected during the first MRI session. If time remained, participants performed 1 or 2 task runs during the first session as well.

### Stimuli and task

Stimuli and task were generated and presented using MATLAB (MathWorks, Natick, MA) in conjunction with Psychtoolbox^57,65^. Memory stimuli consisted of grayscale sinusoidal gratings (diameter 6°, 1 cycle/degree) that had an orientation based on one of three ‘base’ orientations (15°, 75° and 135°). For each trial, base orientations were jittered ± 10°, resulting in 63 possible orientations. Post-testing questioning revealed that no participant was aware that only a subset of all possible orientations was tested. On each trial, two orientations were presented 5.5° of visual angle to the left and right of a central fixation point. Orientation pairs were counterbalanced across trials within runs, and the left and right orientations were never from the same ‘base’ orientation.

Before the start of a trial, the fixation dot turned from white to green or red (depending on the condition) for one second as a pre-trial warning. Simultaneous with the onset of a TR, memory stimuli were presented with a 5 Hz counter-phase flicker for 1,000 ms (see Fig. 1). After offset of the memory stimuli (500 ms interval), a cue appeared for 1,000 ms that indicated which orientation would first be tested, and thus initially attended (‘L’ for left and ‘R’ for right). The color of this cue was congruent with the pre-trial warning color, and indicated whether participants should remember both orientations (RB trials: green cues), and thus store the un-cued item for later use, or whether they should remember that single orientation (RS trials: red cues) and forget the other orientation. This resulted in four different stimulus types: RB-1 and RB-2 cued orientations, versus RS-1 cued and RS-0 orientations.

After a 10-s delay period, participants were presented with a test grating in the same spatial location as the cued memory stimulus, and they indicated whether the test orientation was tilted counter-clockwise or clockwise compared to the remembered orientation. Participants gave their response on an MRI-compatible button box using their right hand (index finger = counter-clockwise, middle finger = clockwise). The test orientation was presented for 2,500 ms, after which on RS trials, the trial ended and the following trial was begun after a jittered (1,500, 4,000 or 6,500 ms) interval. On RB trials, after 1,500 ms the previously unattended orientation was cued (1,000 ms), indicating that participants would now be tested on the second orientation. After another 10-s delay period, a test orientation appeared (2,500 ms) on the same side as the second cued orientation and participants again indicated whether the test orientation was tilted counter-clockwise or clockwise compared to the remembered orientation. The second cued item was always the previously un-cued item. After the second test, the precue for the next trial was presented after a jittered (1,500, 4,000 or 6,500 ms) interval.

Each run contained 24 trials and was counterbalanced on trial type, cue order and “base” orientation pair. This meant that each combination of orientations was presented equally often, avoiding bias in the classification/reconstruction. In addition, the orientations were jittered ± 10°, abolishing any systematic difference between pairs of orientations. Runs started and ended with a 12.5 s grey screen, resulting in 690 s per run, and each participant completed 16–21 runs total. Eyetracking (Avotec Inc) was used to monitor fixation throughout each run.

Performance was staircased at 75% correct discrimination. The orientation difference between the memory and test grating decreased or increased when performance was higher or lower than 75%, respectively, on four consecutive trials of the same memory type. At the end of each run, participants were informed about the degree difference between the memory and test orientation they were discriminating at 75% correct for each memory type separately. Based on the behavioral and pilot results, there was no evidence for a different strategy use between RB-1 and RB-2.

Prior to the fMRI sessions, participants underwent a behavioral training session in which they practiced the task and were trained to keep their eyes on fixation during the entire task run. Eye movements were evaluated using eyetracking (Eyelink-1000, SR Research), and feedback about fixation quality was provided to the participants throughout training.

Because of the small sample and possibly non-normally distributed data, behavioral data were statistically tested using Wilcoxon signed-rank tests between the RB-1, RB-2 and RS-1 trials. In the first experimental MRI run, all participants started with a difference between memory and test of 15°. While we report the analysis of all trials in the main text (Fig. 1), we also repeated the analysis with trials from the final MRI session alone, to examine performance once any learning effects had worn off and behavior had stabilized (Supplementary Fig. 1).

### ROI localization

In the first 2-h scanning session, subjects completed two polar angle retinotopic mapping runs and two stimulus-specific localizer runs to define visual ROIs. For polar angle mapping, a checkerboard wedge (black, white, flickering at 8 Hz) rotated around fixation (one run clockwise and one run counterclockwise; complete revolution in 50 s; 8 repetitions). For stimulus-specific localizer mapping, two sinusoidal gratings that were identical to the memory stimuli flickered at the same locations as the memory stimuli. Six orientations (evenly spaced from 15° to 165°) were presented in a blocked design for 15 s each, followed by a 15 s blank period. The two gratings were always paired such that the absolute difference between the orientations was 60°, just as in the main experiment. The sequence of 6 orientation blocks and 1 blank block was repeated 4 times, and the order of orientation blocks was randomized within each block sequence. Runs started and ended with a 12.5 s blank screen, resulting in a 425-s run. To keep attention focused on the orientations, one or two 45° orientation changes (100-ms) occurred in each 15-s block and participants were asked to press a button every time they detected a brief orientation change.

Initially, our intention was to train the classifier and IEM on the data from the localizer blocks. However, during piloting, we found that classification did not generalize between the localizer and the memory experiment, even during the stimulus presentation period of the memory task. While the reason for this is unclear, it may be at least partially attributable to the simultaneous presentation of two lateralized stimuli, and the interaction with task differences between the localizer and memory task. Previous studies using a perceptual localizer to train a classifier have primarily used a centered presentation of a single stimulus^2,3^. Based on these pilot results, we decided to use the localizer for voxel selection only, and to train the classifier and IEM on epochs of the memory task.

### Preprocessing

Data were analyzed using custom scripts utilizing FSL^66^, AFNI^67^, Freesurfer^68,69^ and MATLAB (MathWorks, Natick, MA). Functional scans were slice-time corrected, despiked, motion corrected and high pass filtered at 0.008 Hz (125 s). All functional scans and the T1-weighted anatomical scan were aligned to the first functional scan of the first session. The anatomical scan was segmented into grey and white matter using Freesurfer, and retinotopic ROIs were then drawn on the resulting inflated cortical surfaces.

### Univariate analysis

First, we performed a univariate analysis on the whole brain during the first delay period to test for sustained delay activity (Fig. 2a). Because we did not have any a priori assumption about the shape of the hemodynamic response function, we implemented a finite impulse response function with a TENT-regressor for each timepoint in the RB and RS trials using AFNI. Beta coefficients for each timepoint were scaled to percent signal change and the estimates for TRs 3–5 were averaged for both the RB and RS trials. This yielded a voxel-wise estimation map for each participant, which was smoothed with an 8-mm FWHM kernel and normalized to 2 mm MNI space before being entered into a one-sample t-test against zero. To assess statistical significance, voxels were first thresholded at a *p* < 0.05. Then, AFNI’s 3dClustSim was used to calculate the minimum number of voxels in a cluster at an α of 0.001 (1,083 voxels).

### ROI analyses

Pilot data from two authors (E.L. and A.V.) suggested that logistic regression classification and IEM reconstruction were most reliable when trained on an ROI of 200 voxels in combined V1, V2 and V3. Therefore, for each hemisphere, we selected the 200 voxels in combined V1, V2 and V3 that were most responsive to the location of a contralateral grating, irrespective of orientation. First, we used standard techniques to draw out early visual areas on the basis of the polar mappings^70^. Then, the de-meaned stimulus-specific localizer data was analyzed using a GLM with a regressor for each orientation block, and six motion regressors. Betas for all orientation blocks were averaged. Based on the average beta, t-values for each voxel in V1–V3 were calculated. Of all voxels in V1–V3, the 200 voxels with the highest t-value were selected for ROI analyses.

In addition, the pilot data showed that averaging the BOLD signal over several TRs made the outcome more robust. Therefore, based on the average BOLD response of all subjects (Fig. 2b), we selected 5 epochs of interest for the RB trials and 3 epochs of interest for the RS trials. Data was z-scored per voxel for each separate run and averaged over the TRs in the epochs of interest. All trial types contained epochs corresponding to the presentation of the memory stimuli (2.5–7.5 s, TR 1–2), the first delay period (7.5–15 s, TR 3–5) and the first test stimulus (15–20 s, TR 6–7), and the RB trials additionally contained epochs for the second delay period (22.5–30 s, TR 9–11) and the second test stimulus (30–35 s, TR 12–13).

### Multi-voxel pattern classification analyses

All fMRI runs were divided into training and test sets using an iterative leave-one-run-out procedure. The training set included all trial types together, irrespective of whether each trial was an RB or RS trial, and irrespective of whether the right or left stimulus was cued first. This resulted in the maximum number of training trials, yielding a more robust analysis. Also, it created an unbiased model with respect to trial type and memory type^71^. Subsequently, testing was performed on each stimulus type separately (see Fig. 3). Training and testing were performed within each hemisphere individually, and then averaged according to stimulus type.

We used penalized logistic regression (L_2_ penalty = 10) implemented in the Princeton MVPA toolbox (https://code.google.com/p/princeton-mvpa-toolbox) to train and test the classifier. For these analyses, the orientations were binned according to the three base orientations such that the classifier had three orientation categories. For each category, a classifier was trained to distinguish that category from the other two categories. To calculate the evidence in favor of the four different stimulus types (RB-1, RB-2, RS-1, RS-0), we subtracted the logistic regression evidence for the orientation that was not presented from the evidence for the contralateral orientation. This subtraction step was necessary because of the large baseline shifts in orientation evidence values between trial epochs in which grating stimuli were present or absent (see Supplementary Fig. S3 for the raw evidence values without this baseline subtraction). We then averaged over trials and hemispheres according to the four stimulus types. To test whether the evidence was different from what would be expected for a null effect (no differentiation between the tested and not-presented orientation), we calculated *p* values using a permutation test. For each participant, training labels were shuffled 1,000 times within runs (while keeping orientation pairings within trials intact) such that there was no relationship between the actually presented/remembered orientations and the orientation labels used for model training. The model trained with permuted data was then tested on the original data, yielding a null distribution of 1,000 mean evidence values for each participant. Then, these values were entered into a second-level analysis across the participants: the set of participant-level mean evidence values were compared to zero with a Wilcoxon signed-rank test, both for the observed data and each of the 1,000 permuted iterations. Next, one-tailed *p* values were calculated by determining the fraction of permuted iterations on which the Wilcoxon signed rank statistic from the permuted data was greater than (right-tailed test) or less than (left-tailed test) the observed signed rank statistic. Finally, a two-tailed *p* value was computed by doubling the most significant one-tailed *p* value. To calculate *p* values for the difference between two stimulus types, the evidence values were subtracted between the conditions of interest for each participant, for both the observed and permuted data, and two-tailed *p* values calculated on the signed rank statistics as before.

Finally, to compare classification performance across cued, un-cued future-relevant, and un-cued future-irrelevant phenomenological WM states, we performed non-parametric one-way repeated measures ANOVAs. Here, we calculated an empirical *F*-value for the true data, as well as *F*-values for each of the 1,000 permuted iterations. Then, the associated one-tailed non-parametric *p* value was calculated as the fraction of null *F*-values that exceeded the observed *F*-value.

### Searchlight analyses

To analyze involvement of areas outside the predefined ROIs, we used a searchlight procedure that systematically examined spherical spatial patterns of voxels throughout the brain^33^. For each participant, we used an individual mask containing only grey matter voxels. We used a kernel of 4 voxels, resulting in patterns of 257 voxels maximum. Since the classifier trained on the delay period gave the statistically most robust results during the delay period, we used this time period to train the same classifier for the searchlight procedure. For this analysis, we did not assume a strictly retinotopic organization, meaning that on each trial trained on the cued stimulus without taking hemisphere into account. We then calculated the evidence for the four different stimulus types by subtracting the evidence for the not-presented orientation from the evidence for the cued and un-cued/discarded items in each trial. These four evidence maps were calculated for each participant and normalized to 2 mm MNI space. A group-level t-test against 0 was performed to identify voxels that showed significant evidence (voxelwise *p* < 0.05) above or below baseline. Cluster thresholding was based on determining the minimum number of voxels in a mask at an α of 0.001 by using 3dClustSim. This resulted in a minimum of 428 voxels per cluster to survive cluster thresholding. This procedure was repeated for each memory type, resulting in four maps corresponding to RB-1, RB-2, RS-1 and RS-0.

### Inverted encoding model reconstructions

For the IEM, the same training and test sets were used as for the classification. First, to remove the non-orientation-selective baseline shifts observed in the classification analysis between trial epochs with and without stimuli on the screen (Supplementary Fig. S3), we z-scored each voxel’s response across trials, separately within the training and test sets on each cross-validation.

To reconstruct the presented/remembered orientation during different parts of the trial, we used an IEM approach similar to that implemented by Brouwer and Heeger^29^. The orientation selectivity of each voxel in the ROI was defined as the weighted sum of nine hypothetical orientation channels. Hypothetical channels were half—wave-rectified sinusoids raised to the ninth power, and the nine channels were distributed evenly from 0–179°. The weights of the channels were estimated based on the training sets using linear regression. Let *B*_*1*_ (*m* voxels × *n* trials) be the observed signal in the training set for each original orientation (from 0–179°), *C*_*1*_ (*k* channels × *n* trials) be a matrix of predicted responses based on the hypothetical channels for that orientation, and *W* (*m* voxels × *k* channels) be the estimated weights.

$$ W = B_{1} \times C_{1}^{T} \times \, \left( {C_{1} \times \, C^{T} } \right)^{ - 1} $$

These weights were then used to reconstruct the channel outputs (*C*_2_) associated with the activity on the test run for each original orientation (*B*_2_).

$$ C_{2} = \left( {W^{T} \times \, W} \right)^{ - 1} \times W^{T} \times B_{2} $$

Finally, we created trial-wise orientation reconstruction functions by calculating a weighted sum of the hypothetical orientation channels, weighted by the calculated channel response magnitudes (*C*_2_). The peak of this reconstruction therefore corresponded to the orientation that was most likely to be represented on that trial. To evaluate these reconstructions, all curves were re-centered so that 0 corresponded to the actually presented/remembered orientation, and averaged across trials within each participant and condition. The reliability of each reconstruction was then calculated with a “representational fidelity” metric *RF* (as has been done previously, i.e.^42,72^). For each participant and condition, we calculated the vector mean across the orientation reconstruction, as shown below, where r(θ) is the weighted channel activation at a given polar angle, spanning all − 90° to 89° of zero-centered orientation space.

$$ RF = mean\left( {{\text{r}}\left(\uptheta \right)\cos 2 \times\uptheta } \right) $$

Next, we combined these results across participants by calculating the mean *RF* value in each condition. Then, to assess whether the observed reconstructions deviated from those expected by chance, we created a null distribution of representational fidelity values by repeating the above IEM analysis for 1,000 permutations of the data, as described in the *Multi-Voxel Pattern Classification Analysis* section. Finally, we calculated a two-tailed empirical *p* value for each condition by calculating the fraction of samples in the null distribution whose absolute value exceeded the absolute value of the observed mean representational fidelity value.

### Simulations

Simulations were performed to examine potential mechanisms that could explain the below-baseline evidence for information discarded from WM. The simulations were based on the IEM as described above. Six participants were simulated (matching the sample size), each of whose data consisted of 200 voxels with a random weight distribution across the channels drawn from a uniform distribution. For each participant, training data were generated through the equation:

$$ B_{train} = W \times C_{train} + \varepsilon_{BOLD} $$

where *W* (*m* voxels × *k* channels) is a matrix of channel weights, *C*_*train*_ (*k* channels × *n* trials) is a matrix of channel responses, and *ε*_*BOLD*_ is Gaussian noise. The weight matrix, *W*, was drawn from a random uniform distribution and normalized such that the weights of a given voxel summed to 1. On each training trial, the model was presented with a single orientation from which the channel response was determined. Orientations were drawn from the same distribution of the experiment such that the simulation training had most direct correspondence to training on the stimulus phase in the experiment. The noise level was set to produce a signal-to-noise ratio of 0.7. Varying this parameter within reasonable ranges produces quantitative, but not qualitative changes in the simulations.

*C*_*train*_ was computed by:

$$ C_{train} = M_{train} \times \left( {\varphi_{train} \times S_{train} + \varepsilon_{neural} } \right) $$

where *M*_*train*_ is the (*k* channels × 180 degrees) model of idealized half-wave rectified tuning channels raised to the eighth power identical to those used in the IEM above. φ_train_ is a scaling parameter controlling the strength of the stimulus, which was set to 1, because the training data mimics training on the stimulus presentation period. *S*_*train*_ is a 180 degree Gaussian stimulus vector formed by convolving a Kroenecker delta function centered at the presented angle with a Gaussian with an amplitude of 1 and standard deviation of 18 degrees. Modeling the stimulus input as a Gaussian rather than a delta function admits some degree of sensory uncertainty, as has been previously observed^73^. *ε*_*neural*_ is Gaussian “neural” noise with an amplitude of 0.2 and standard deviation of 0.05. *C*_*train*_ was rectified to reflect a simulated firing rate with a floor of 0. Adjusting arbitrarily defined parameters within reasonable ranges results in quantitative, but not qualitative changes to the simulations.

On each testing trial, we randomly selected an orientation, *Θ*_*sample*_, using the same orientation sampling utilized in the experiment. For reference, we also designated a held out orientation, *Θ*_*held*_, in the same manner of the experiment. The testing data were computed by:

$$ B_{test} = W \times C_{test} + \varepsilon_{BOLD} $$

and

$$ C_{test} = M_{test} \times \left( {\varphi_{test} \times S_{test} + \varepsilon_{neural} } \right) $$

*M*_*test*_ corresponded to a “biased” version of *M*_*train*_. These biases reflect parametric changes to gain, width, and preferred stimulus of the receptive fields that have been observed in vivo^36,37^, and proposed to lead to behavioral after-effects^38,39,51^. The simulations tested whether such modulations could account for the changes in representation observed during cued, un-cued future-relevant and un-cued future-irrelevant sates. In models including a change in gain, the amplitude of tuning channels was increased or decreased as a function of distance to *Θ*_*sample*_ (see Fig. 7b). In models including a change in width, the width of tuning channels was narrowed or widened as a function of distance to *Θ*_*sample*_. In models including a shift, the preferred orientation of tuning channels was shifted towards *Θ*_*sample*_, as a function of distance to *Θ*_*sample*_. Let c be a half-wave rectified sine curve reflecting the idealized tuning response. A change in gain was accomplished by scaling *C*_*test*_ by α, a change in width was accomplished by raising *C*_*test*_ to the β power, and a shift was accomplished by moving the center of *c* by δ. Following (^39^):

$$ \alpha = 1{-}\gamma \times \left( {1 + \cos 2\theta } \right) $$

and

$$ \beta = 8 \times e^{[1 - \mu (1 - 2\cos 2\theta )]} $$

Here, γ and μ control the strength of gain and width tuning, respectively. Finally, following (^35^):

$$ \delta = \frac{\omega d}{{1 + e^{{ - a\left( {d - b} \right)}} }} $$

Hence, shifting was a sigmoidal function of distance, *d*, of a given channels’ center to *Θ*_*sample*_, with strength proportional to ω. *a* and *b* were set to − 0.1 and 20, respectively.

*M*_*train*_ is identical to *M*_*test*_ with the model parameters γ, μ, and ω set to 0.

Finally, *S*_*test*_ was computed in the same manner as *S*_*train*_ and scaled by φ_test_. In this case, φ_test_ can be conceived of as memory strength and approximates neural firing. If set to 1, φ_test_ is identical to φ_train_ which mimics training and testing during stimulus presentation. Reductions of φ_test_ simulate reductions in memory strength that may occur with the passage of time. To mimic the experimental design and analyses, the simulated training data and simulated testing data consisted of 18 runs of 24 trials each. Training and testing data were matched such that each testing run had the same stimuli as a corresponding training run with independent noise. Hence, a given run of testing data could function as a later part of the corresponding training data. Because there was no evidence for reliable ipsilateral stimulus or memory representations in the experimental data (see Supplementary Fig. S8), only one representation per trial period was modeled. To match the leave-1-run-out analysis performed on the experimental data, an IEM was trained on all but 1 of the training runs, and tested on the held-out run. Training and testing data were z-scored independently on a voxel-by-voxel basis prior to training and testing. Orientation reconstructions were computed using the method as described in *Inverted Encoding Model*. Results were averaged across trials within a simulated participant, and then across simulated participants.

To determine appropriate values of γ, μ, ω, and φ_test_, the simulated data were fit to the experimental data separately for each representational state. Representational states consisted of the stimulus phase, cued item, un-cued future-relevant item, and un-cued future-irrelevant item. Parameters were constrained to the following ranges: − 1 ≤ γ ≤ 1, − 1 ≤ μ ≤ 1, 0 ≤ ω ≤ 1, and 0 ≤ φ_test_ ≤ 1. To reduce over-fitting, both the state-of-interest, as well as the comparable state for the held out orientation (either stimulus phase or delay phase) were simultaneously fit. Fitting was done in multiple steps. First, a grid search of the parameter space was performed by independently varying each parameter in 11 steps through its range using sum squared error of the fit as a cost function. The best parameter vector as determined by the sum squared error of the model fit was then used as a starting point for more comprehensive model space exploration using Bayesian Adaptive Direct Search (BADS^74^) which allows effective exploration of model spaces with noisy objective functions. To determine whether modulations of receptive field tuning parameters were needed to fit the data, separate fits were also performed fixing one or more of γ, μ, and ω to 0.

Model assessment was performed with respect to the representational state of interest. F-tests were used to compare nested models in order to determine whether the increase in explained variance justified inclusion of modulatory parameters. For these comparisons, the degrees of freedom were estimated to be 24 corresponding to the 8 tuning curves, each described by a center, height, and width. Across all models, 6 parameters were fixed (*ε*_*BOLD*_*, ε*_*neural*_ amplitude, *ε*_*neural*_ s.d., stimulus uncertainty, *a*, and *b*), and φ_test_ was free to vary. Hence, model comparisons accounted for additional degrees of freedom used by including γ, μ, and/or ω. In addition to these model comparison statistics, fits were assessed by explained variance, and adherence of the simulated data to within a standard error of the experimental data.

## Supplementary information

Supplementary file1

## Data Availability

All fMRI data and code will be shared upon request. Simulation code is publicly available at: https://osf.io/cq35b/.
