# Supplementary material for: Dissociable neural mechanisms underlie currently-relevant, future-relevant, and discarded working memory representations
Source: Sci Rep. 2020 Jul 8;10:11195. doi: 10.1038/s41598-020-67634-x (PMC7343803; doi:10.1038/s41598-020-67634-x)
Supplement: Supplementary file 1 — Supplementary file1 [file 41598_2020_67634_MOESM1_ESM.docx]

**Dissociable neural mechanisms underlie currently-relevant, future-relevant, and discarded working memory representations**

*Elizabeth S. Lorenc*, Annelinde R.E. Vandenbroucke*, Derek E. Nee, Floris P. de Lange, Mark D’Esposito*

**Supplementary Materials**

***Supplementary figure S1.*** Orientation differences discriminated by all participants in their final MRI sessions (scan day 4). All comparisons between conditions *p* = 0.03 with Wilcoxon signed rank tests, do not pass Bonferroni-corrected *α ­=* 0.017.

**
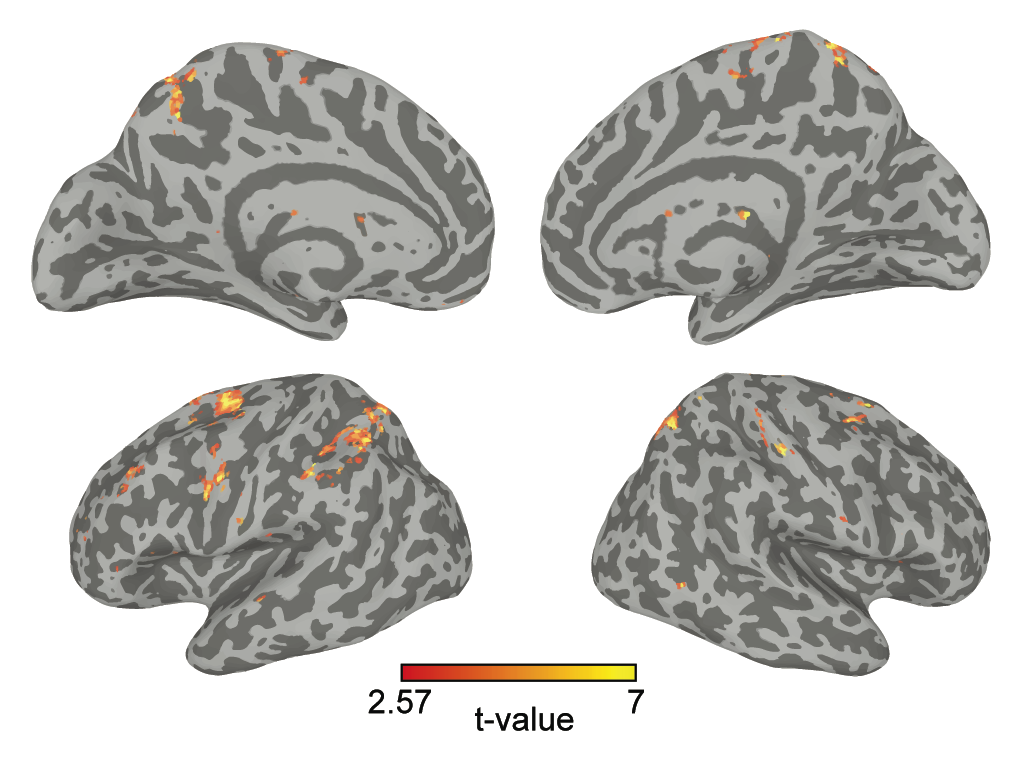
**

***Supplementary figure S2****.* Univariate whole brain analysis of mean activity during the first delay period, voxel-wise family-wise error corrected at *p* < 0.05 without cluster correction.

**

***Supplementary figure S3.*** Raw evidence values associated with the early visual cortex classification results in Figure 3 in the main manuscript (i.e. without subtracting the evidence for the not-presented orientation category as a baseline). Classification evidence based on training on data from the stimulus presentation period (**a**) and the first delay period (**b**). The large baseline shifts in raw evidence values, both between trial epochs and across trial types, underscore the need to report results relative to a properly-matched baseline. Shaded areas denote between-subject standard error.

**

***Supplementary figure S4****.* Early visual cortex classification outcome when testing each volume separately. Classification training for each volume is based on the stimulus presentation period (**a**) and the first delay period (**b**; see Figure 1 for abbreviations). Shaded areas denote between-subject standard error.

**

***Supplementary figure S5.*** Early visual cortex decoding analyses for each subject (separate markers) plotted separately along with the average (colored solid lines). Classification based on training on data from the stimulus presentation period (**a**) and the first delay period (**b**; see Figure 1 for abbreviations). Shaded areas denote between-subject standard error.

***Supplementary figure 6.*** Early visual cortex classification results when trained only on RB-1 and RS-1 items during the delay period. Shaded areas denote between-subject standard error.


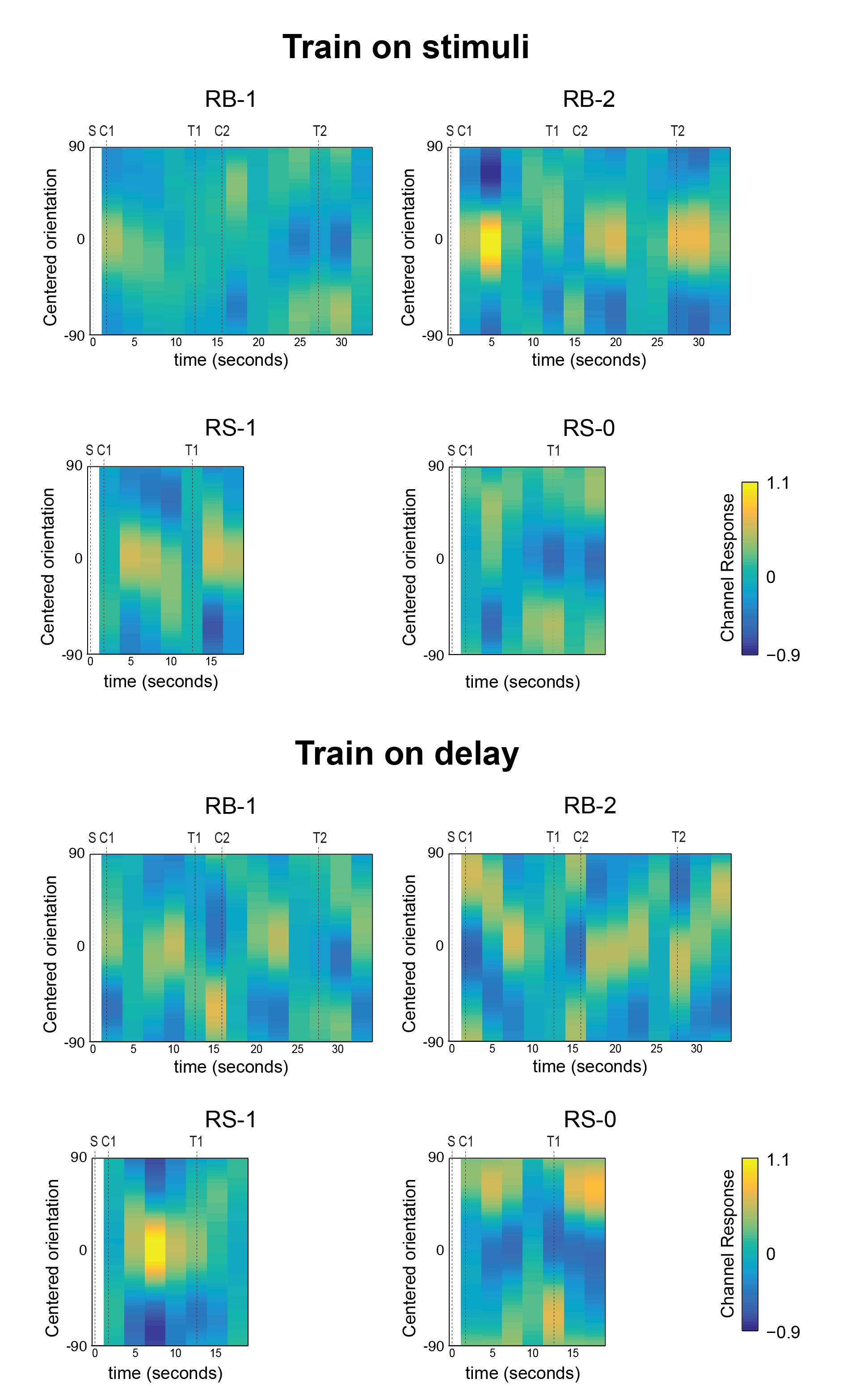


***Supplementary figure 7****.* Early visual cortex reconstruction analyses when testing each volume separately. IEM training for each volume is based on the stimulus presentation period (**a**) and the first delay period (**b**; see Figure 1 for abbreviations). Colors denote the reconstructed channel response functions, calculated as the sum of all orientation channels, weighted by their reconstructed activation levels.

***Supplementary figure 8.*** Early visual cortex orientation reconstruction for each subject (rows) and each stimulus type (columns) for the five time periods of interest in the main analyses. Classification based on training with the stimulus presentation period (**a**) and the first delay period (**b**; see Figure 1 for abbreviations). Colors denote the reconstructed channel response functions, calculated as the sum of all orientation channels, weighted by their reconstructed activation levels.


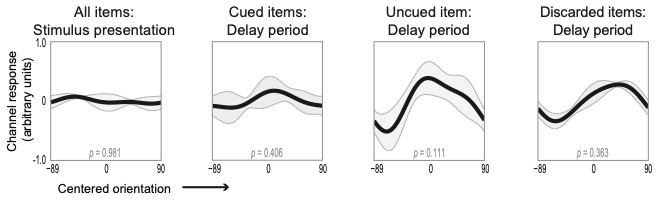


***Supplementary figure 9.*** Early visual cortex orientation reconstruction for the ipsilateral stimulus presentation. We performed this analysis to determine whether there was any evidence of ipsilateral encoding or maintenance that should be taken into consideration for the computational modeling. ‘All items: stimulus presentation’ represents the reconstruction averaged over all items; ‘cued items: delay period’ represents reconstruction averaged over RB-1 and RS-1, Delay 1, and RB-2, Delay 2; ‘un-cued item: delay period’ represents the reconstruction of RB-2 during Delay 1; ‘discarded items: delay period’ represents the reconstruction averaged over RB-0, Delay 1, and RB-1, Delay 2. Based on these reconstructions, we decided not to include the ipsilateral stimulus in the computational modeling analyses.
